# Human macrophages activate a coordinated program linking inflammation and cell death in response to intracellular Poly(I:C)

**DOI:** 10.3389/fimmu.2026.1827486

**Published:** 2026-10-02

**Authors:** Luigi Fiore, Lara Di Leonardo, Marco Buscetta, Maria Concetta Volpe, Marta Cristaldi, Maria Rita Giuffrè, Chiara Finocchio, Gioacchin Iannolo, Germana Lentini, Giuseppe Teti, Alice Conigliaro, Harald Husebye, Concetta Beninati, Chiara Cipollina

**Affiliations:** 1 Ri.MED Foundation, Palermo, Italy; 2 Dipartimento di Scienze Biomediche, Odontoiatriche e Delle Immagini Morfologiche e Funzionali, Università Degli Studi di Messina, Messina, Italy; 3 Centre of Molecular Inflammation Research, Department of Clinical Molecular Medicine, Norwegian University of Science and Technology, Trondheim, Norway; 4 Dipartimento di Biomedicina, Neuroscienze e Diagnostica Avanzata, Università Degli Studi di Palermo, Palermo, Italy; 5 IRCCS ISMETT (Istituto Mediterraneo per i Trapianti e Terapie ad Alta Specializzazione), Palermo, Italy; 6 Department of Medicine and Surgery, Università Degli Studi di Enna “Kore”, Enna, Italy; 7 Dipartimento di Patologia Umana, Università Degli Studi di Messina, Messina, Italy; 8 Scylla Biotech Srl, Messina, Italy

**Keywords:** caspase, cell death, cellular immune response, cytokine, double stranded RNA (dsRNA), inflammation, interferon, macrophage

## Abstract

**Background:**

Macrophages sense viral double-stranded RNA (dsRNA) via the innate immune receptors TLR3 and MDA5 whose activation drives a robust inflammatory program, often coupled to cell death. TLR3/TRIF-dependent apoptosis has been described in response to dsRNA. Growing evidence suggests that dsRNA may trigger multiple forms of cell death with mechanisms yet to be elucidated. In addition to TLR/TRIF axis, the necroptosis regulator RIPK3 has been implicated in both inflammatory response and cell death during viral infections in several cell types. However, its role in regulating these two events in human macrophages remains unclear.

**Methods:**

Here, we investigated in human monocyte-derived macrophages (hMDMs) the mechanisms of dsRNA induced cell death and its temporal and molecular linkage to inflammatory signaling, with a focus on the interplay between RIPK3 and the TLR3/TRIF pathways.

**Results:**

We found that intracellular Poly(I:C), a synthetic analogue of dsRNA, induced cell death with apoptotic and necroptotic features. TRIF-mediated signaling cascade and RIPK3 jointly contributed to caspase-8 activation leading to apoptosis. RIPK3 also promoted necroptosis by inducing MLKL phosphorylation. Downstream of caspases, Poly(I:C) induced cleavage of Gasdermin E (GSDME), which remained intracellular and localized in mitochondria contributing to the amplification of caspase-3 activation. RIPK3-dependent signaling drove the release of IFN-β and TNF-α and promoted the upregulation of the sensors TLR3 and MDA5, thereby amplifying the inflammatory axis. The inflammatory response preceded cell death, with RIPK3 being the key regulator of the early signaling and TLR3/TRIF becoming involved at later timepoints.

**Conclusions:**

Collectively, these findings identify RIPK3 as key mediator coordinating dsRNA-induced inflammatory signaling and regulated cell death in human macrophages.

## Graphical Abstract

**Figure:**
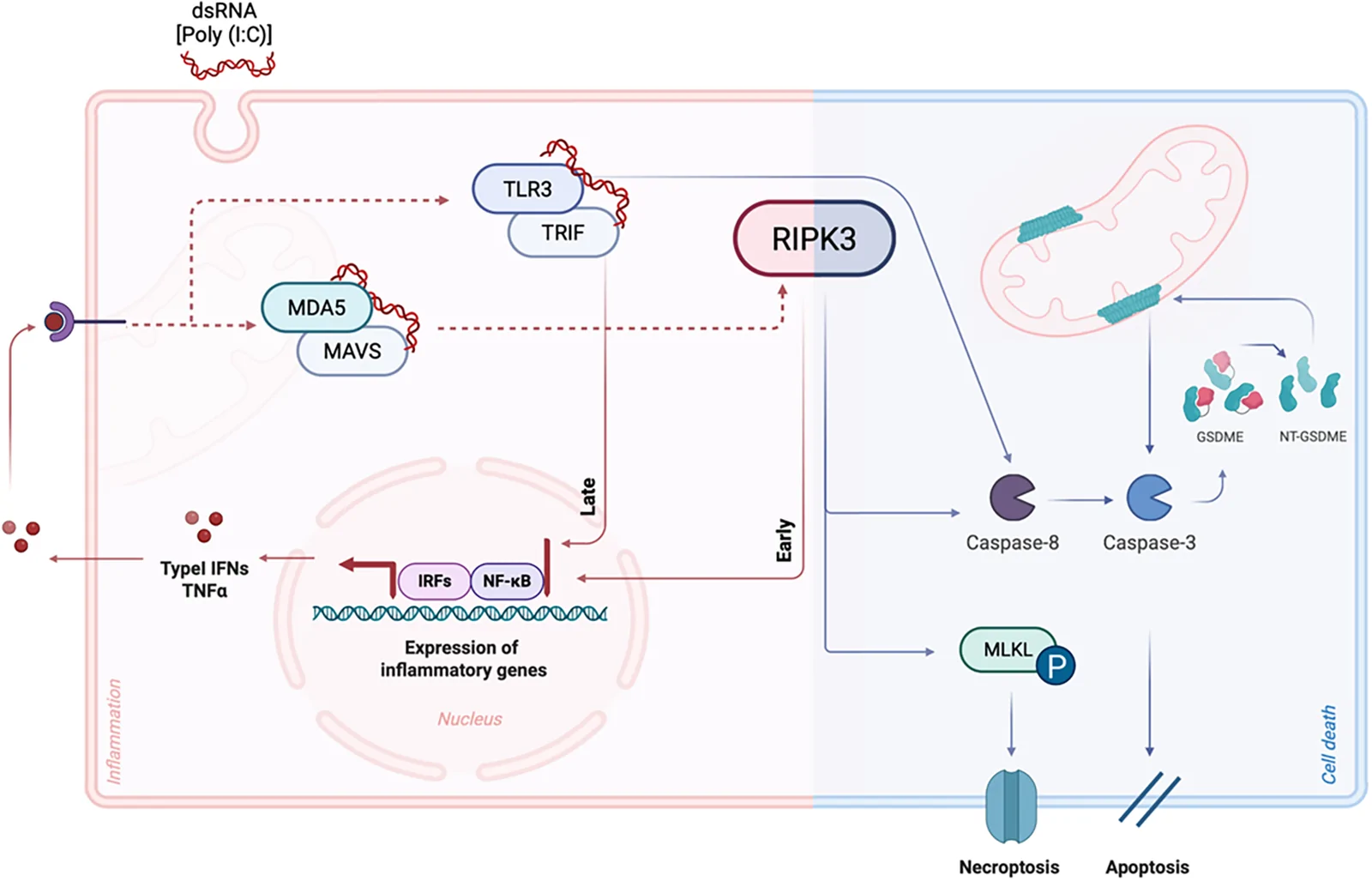
RIPK3 coordinates inflammation and cell death in response to intracellular Poly(I:C) in human macrophages. Upon cell entrance, Poly(I:C) activates RIPK3 dependent inflammatory responses possibly via MDA5/MAVS signaling or other upstream regulators (dashed lines). RIPK3 promotes Type I IFN and pro-inflammatory cytokine release. TLR3/TRIF becomes involved in the control of the inflammatory response at later time points. Release of IFNs promotes the upregulation of MDA5 and TLR3. Together with TLR3/TRIF, RIPK3 is also responsible for controlling the activation of caspase-8/-3 axis which leads to GSDME cleavage. GSDME-NT localizes in the mitochondria amplifying caspase-3 activation. At the same time, RIPK3 promotes necroptosis by phosphorylating MLKL. Created in BioRender. Conigliaro, A. (2026) https://BioRender.com/spnttq1.

## Introduction

Viral infections represent a major global health challenge and are a leading cause of acute and chronic airways diseases. Despite advances in prevention and treatment, many viral infections remain difficult to manage (1, 2). A better understanding of the infection process, from early detection to resolution, is crucial for preventing complications. Innate immune cells, especially resident macrophages, are essential for initiating and coordinating frontline protective responses (3–5). Recognition of viruses reaching the airways occurs via detection of pathogen associated molecular patterns (PAMPs) by innate immune receptors including Toll-like receptors (TLRs) (6), nucleotide-binding and oligomerization domain (NOD)-like receptors (NLRs) (7), and retinoic acid-inducible gene I (RIG-I)-like receptors (RLRs) (6, 7). As soon as viral PAMPs are recognized, the inflammatory response is activated through the release of cytokines and chemokines leading to the recruitment of other immune cells to mount an effective response aimed at eliminating the pathogen and restoring homeostatic conditions (8, 9). RNA viruses induce antiviral Type I Interferon (IFN) response in infected cells (10). Following their internalization, viruses release their genomic RNA into the cytoplasm. This starts the replication process, leading to the formation of double-stranded RNA (dsRNA), generated as an intermediate product (11). Viral dsRNA is sensed by pattern recognition receptors such as TLR3 and RLRs (12, 13). TLR3 is a membrane spanning receptor mostly located within endosomes. RLRs are intracellular receptors among which RIG-I and melanoma differentiation-associated protein 5 (MDA-5) have been reported to preferentially recognize different type of dsRNA, with MDA5 being the main receptor for the synthetic dsRNA Poly(I:C) (13–15). The binding of dsRNA to TLR3 triggers the activation of TIR-domain-containing adapter-inducing interferon-β (TRIF) signaling cascade while RLRs signal via mitochondrial antiviral signaling (MAVS)-pathway (16–20). Both cascades converge towards the activation of the transcriptional factors IRF-3 (16–20) and NF-kB (12, 19) which control the upregulation of Type I IFN genes (10) and proinflammatory cytokines (i.e. TNF), respectively (21, 22). Limited evidence suggests that the necroptotic regulator RIPK3 may also contribute to control the inflammatory response downstream of MDA5/MAVS signalling axis during viral infections (23). However, what is the role of RIPK3 in controlling cytokine release following Poly(I:C) transfection as well as what is its relation with TLR3/TRIF signalling axis remains to be clarified.

In addition to mount massive inflammatory responses, infected cells may undergo programmed cell death (PCD) as a mechanism of defence (24). Several reports have shown that dsRNA can induce the activation of the apoptotic cascade triggered by TLR3/TRIF/Caspase-8 axis (25–27). Apoptosis has been generally recognized as an immunogenic silent form of PCD triggered following the activation of the apical apoptotic caspases (either caspase-8, or caspase-9), which in turn cleave and activate the executioner caspase-3/-7 (28–31). Recent evidence has shown that caspase-3 is able to cleave and activate the pore-forming protein Gasdermin E (GSDME) and this may turn apoptosis into highly immunogenic pyroptosis with mechanisms yet to be defined (32–34). Necroptosis has also been reported to occur in response to viral infection, with RIPK3 being a key orchestrator of the cell death cascade (35). However, what is the role of RIPK3 in Poly(I:C)-induced macrophage death and what is the connection between Poly(I:C)-induced inflammatory response and regulated cell death has never been explored.

Herein we investigated the crosstalk between inflammation and cell death in human primary macrophages exposed to the dsRNA mimetic Poly(I:C). We identified RIPK3 as the key regulator of the early inflammatory response to Poly(I:C) with the TLR3/TRIF axis becoming involved at later timepoints. RIPK3-dependent signaling appeared to be dominant over the TLR3/TRIF axis in the control of the inflammatory response. On the contrary, both RIPK3 and TRIF-mediated pathways jointly contributed to caspase-8 activation and apoptosis, which was followed by GSDME cleavage. RIPK3 also controlled the activation of the necroptotic cascade thus emerging as key regulator of inflammation and regulated cell death in response to Poly(I:C).

## Materials and methods

### Reagents

RPMI 1640 medium (ECB9006L), L-glutamine (ECB3000D), foetal bovine serum (FBS) new cat. (ECS5000L), sodium pyruvate (ECM0542D), HEPES (ECM0180D) and penicillin–streptomycin (ECB3001D) were purchased from Euroclone (Milan, Italy). Human M-CSF (130–096–492) was purchased from Miltenyi Biotec (Bergisch Gladbach, Germany). LPS from Escherichia Coli O111:B4 (L3012), Nigericin sodium salt (N7143), Z-VAD-FMK pan-caspase inhibitor I (627610), GSK872 RIPK3 inhibitor (Hy-101872), Necrostatin-1 RIPK1 inhibitor (N9037), CP-456773 sodium salt (MCC950) NLRP3 inhibitor (PZ0280) and Triton X-100 (9002-93-1) were purchased from Sigma-Aldrich (St. Louis, MO, USA). Pepinh-TRIF inhibitor (tlrl-pitrif) was purchased from InvivoGen (San Diego, CA, USA). Poly(I:C) high molecular weight (tlrl-pic) was purchased from InvivoGen (San Diego, CA, USA). JC-1 mitochondrial membrane potential probe (T3168) and SYTOX™ Green nucleic acid stain (S7020) were purchased from Thermo Fisher Scientific (Waltham, MA, USA). FuGENE^®^ transfection reagent (E2311) was purchased from Promega (Madison, WI, USA). Incucyte^®^ Caspase-3/7 Dye for Apoptosis (4704) was purchased from Sartorius (Essen BioScience, Ann Arbor, MI, USA). Primary antibodies used for western blot included β-actin (sc-81178, Mouse Monoclonal Antibody, 137CT26.1.1) from Santa Cruz Biotechnology (Dallas, TX, USA); β-tubulin (2146, Polyclonal Antibody), phospho-MLKL (D6H3V, Rabbit Monoclonal Antibody #91689), MLKL (D2I6N, Rabbit Monoclonal Antibody #14993), full-length caspase-3 (9662S, Polyclonal Antibody), Cleaved Caspase-3 (9661S) and GSDMD (E8G3F, Rabbit Monoclonal Antibody #97558) from Cell Signaling Technology (Danvers, MA, USA); Caspase-8 (12F5, Mouse Monoclonal Antibody ALX-804-242) from Enzo Life Sciences (Farmingdale, NY, USA); and GSDME (ab215191, Rabbit Monoclonal Antibody EPR19859) from Abcam (Cambridge, UK). Primary antibodies were used at the following dilutions: anti-actin 1:10000, anti-β-tubulin 1:10000, anti-p-MLKL 1:1000, anti-MLKL 1:1000, anti-caspase-8 1:500, anti-full-length caspase-3 1:1000, anti-cleaved caspase-3 1:500, anti-GSDME 1:1000 and anti-GSDMD 1:1000. Secondary antibodies goat anti-mouse IRDye 680RD (926-68070) and donkey anti-rabbit IRDye 800CW (926-32213) were purchased from LI-COR (Lincoln, NE, USA) and used at a dilution of 1:5000. Primary antibodies for immunofluorescence included GSDME-NT (E8G4U, Rabbit Monoclonal Antibody #55879, 1:100) from Cell Signaling Technology (Danvers, MA, USA) and TOM20 (MABT166, Mouse Monoclonal Antibody, 2F8.1, 1:500) from Sigma-Aldrich (St. Louis, MO, USA). Donkey anti-rabbit IgG Alexa Fluor 488 (A21206) was purchased from Invitrogen (Thermo Fisher Scientific, Waltham, MA, USA). CellMask™ plasma membrane stain (C10046) was purchased from Thermo Fisher Scientific (Waltham, MA, USA) and Hoechst 33342 from ImmunoChemistry Technologies (Bloomington, MN, USA).

### Human monocyte-derived macrophages

Peripheral blood mononuclear cells (PBMCs) were isolated from buffy coats obtained from healthy donors and supplied by ARNAS “Civico, Di Cristina, Benfratelli” (Palermo, Italy), in accordance with a Material Transfer Agreement executed on January 7th, 2025. Human macrophages were obtained by culturing PBMCs for 7 days in a complete RPMI 1640 medium supplemented with 10% FBS and 50 ng/ml of human M-CSF. The medium was replaced after 3 days of culture. Macrophage purity and differentiation status were assessed by multiparametric flow cytometry (**Supplementary Figure 1**). The day before each experiment, human monocyte-derived macrophages (hMDMs) were treated with Tryple for 10 min, scraped, plated in complete medium into 96-well plates (5 × 10^4^ cells/well), 12-well plates (7 × 10^5^ cells/well), or 6-well plates (1.5 × 10^6^ cells/well), and incubated at 37 °C, with 5% CO2.

The day of the experiment, culture medium was changed to 1% FBS and cells were stimulated for 18h, unless otherwise indicated, with 1 μg/mL for transfected Poly(I:C) and 10 μg/mL for non-transfected Poly(I:C). Poly(I:C) was transfected with FuGENE^®^ HD Transfection Reagent (Promega) in according to the manufacturer’s protocol. Poly(I:C) concentrations were selected based on preliminary dose-response experiments (**Supplementary Figure 2**) showing that in the case of transfected Poly(I:C), LDH and IFNβ release reached a maximum at 1 μg/ml and did not further increase when stimulating hMDMs with 10 or 100 μg/ml Poly(I:C). Therefore, 1 μg/ml was selected as the optimal concentration for further experiments. In the case of non-transfected Poly(I:C), we found no induction of cell death (LDH release) nor IFNβ release at all tested concentrations. In this case, 10 μg/ml Poly(I:C) was selected because it represented an intermediate-to-high stimulation setting. FuGENE^®^ HD Transfection Reagent (Promega) was selected because it offered very high transfection performance with minimal impact on cell pathways associated with immune-related functions (36). Where indicated, cells were pretreated for 1 h with the indicated inhibitors, including Pepinh-TRIF (25 μM), MCC950 (1 μM), Z-VAD-fmk (20 μM), Necrostatin-1 (50 μM), and GSK872 (10 μM), alone or in combination, prior to Poly(I:C) stimulation.

### THP-1 cells and GSDME silencing

The human monocytic cell line THP-1 (ATCC TIB-202) was grown in complete RPMI 1640 medium supplemented with 10% FBS and differentiated with phorbol 12-myristate 13-acetate (PMA, 5 ng/mL) for 48 hours. On the day of stimulation, culture medium was changed to 1% FBS and cells were stimulated using the same protocol as for hMDMs.

GSDME silencing was achieved using a lentiviral vector (DFNA5 Human shRNA Plasmid Kit (Locus ID 1687), TL313499, Origene Technologies), as previously described (37). Briefly, HEK 293T were transfected with the lentiviral vector and the two helper plasmids (38). After 72 hours, supernatants were centrifuged (800g 10’ RT), filtered (0.45 µM) and used to infect non-differentiated THP-1 cells. 10–12 days after infection cells were sorted for GFP and then silencing efficiency was validated by western blot.

### Isolation of total RNA and real-time quantitative PCR

Total RNA was extracted using TRIzol Reagent (Invitrogen, Carlsbad, CA), following the manufacturers’ instruction. For reverse transcription, the equivalent volume to 1 μg RNA was reverse-transcribed to cDNA, using the iScript cDNA Synthesis kit (Bio-Rad, Hercules, CA, USA). Realtime quantitative PCR was carried out on Step One Plus Real-time PCR System (Applied Biosystems, Foster City, CA, USA) using specific FAM-labeled probe and primers validated. TaqMan Gene expression assay for IFN-β (Hs01077958-s1), TNF-α (Hs99999043-m1), NLRP3 (Hs00918082-m1) and IL-1β (Hs01555410-m1) Applied Biosystems. Gene expression was normalized to GAPDH (prevalidated TaqMan Gene expression assay for GAPDH, Hs03929097g1, Applied Biosystems) endogenous control gene. Relative quantification of mRNA was carried out with the comparative CT method (2^−ΔΔCT^) and was plotted as a relative fold-change compared to untreated cells that were chosen as the reference samples.

### Cell viability assay - lactate dehydrogenase assay

At the indicated time, culture supernatants were transferred into a new 96-well plate and the LDH released from cells was measured using the CytoTox 96R Non-Radioactive Cytotoxicity Assay LDH Cytotoxicity Assay Kit (G1780, Promega) according to the manufacturer’s protocol. Absorbance was measured using a Spark (Tecan) plate reader at wavelengths of 490 nm.

### Western blot

Cells were lysed using RIPA buffer (89901, Thermo Scientific) supplemented with protease inhibitor (P8340, Sigma-Aldrich) and phosphatase inhibitor (P0044, Sigma-Aldrich). Total protein concentration was determined using the BCA protein assay kit (23225, Thermo Scientific). Equal amounts of protein (30 μg) were resolved by SDS–PAGE and transferred onto nitrocellulose membranes (Bio-Rad). For supernatant precipitation, cells were stimulated in the medium with 1% FBS, and samples were processed as previously described (39). Membranes were incubated overnight with primary antibodies at 4 °C, followed by incubation with fluorescent secondary antibodies. Protein bands were detected and analyzed using the Odyssey Imaging System (LI-COR).

### ELISA assay

IFN-β (DY814-05), TNF-α (DY210), S100A8/A9 (DY8226) Galectin-1 (DY1152) and Galectin-3 (DY1154) were measured in culture supernatants using ELISA kit (R&D Systems, Minneapolis, MN) following the manufacturers’ instructions.

### Fluorescence microscopy

Cells were seeded in 96-well plates (PhenoPlate 96-well, black, optically clear flat-bottom, Revvity, Waltham, Massachusetts, USA). After stimulation, cells were incubated with 10 μM of JC1 dye + 1.5 μg/ml of Hoechst 33342 in PBS for 15’ at 37 °C, and then gently washed with PBS. Image acquisition was performed using Operetta CLS (Revvity) with a 63X objective. Nuclear DNA condensation was measured by Harmony Software (Revvity) as mean fluorescence intensity (MFI) and nuclear area (µm^2^) of the nucleus stain Mitochondrial membrane potential, indicated by the red (485/590nm)/green (485/529nm) fluorescence intensity ratio was measured by TECAN plate reader. For GSDME-NT localization analysis, after stimulation, cells were fixed in paraformaldehyde/phosphate-buffered saline (PFA) 4% for 15′ at room temperature (RT), permeabilized with 0.1% Triton X-100 for 15′ at RT, blocked with 5% bovine serum albumin (BSA) in 0.1% Triton X-100 for 1h at RT. Cell were incubated with anti-GSDME-NT primary antibody (1:200, over-night, 4 °C) followed by secondary antibody donkey anti-rabbit IgG Alexa Fluor 488 (1:500, 90′ at RT). For membrane staining CellMask was used, after secondary antibody incubation (1:5000, 30’). Hoechst staining solution (1:5000, 10′ at RT). Image acquisition was performed using Operetta CLS (Revvity) with a 63× objective.

For assessment of plasma membrane permeabilization, SYTOX™ Green dye was added to the culture medium according to the manufacturer’s instructions (30 nM). Membrane permeability was monitored in real time using the IncuCyte system, with images acquired every 15 min. Quantification was performed using the integrated IncuCyte software.

For real-time analysis of caspase-3/7 activation, the IncuCyte^®^ Caspase-3/7 Red Apoptosis Assay Reagent (500 µM) was added to the culture medium according to the manufacturer’s instructions. Cells were placed in the IncuCyte live-cell analysis system and monitored for 24 h, with image acquisition performed every hour.

### Statistical analysis

Statistical analysis was performed using GraphPad Prism 9.0.1 (GraphPad Software Inc., San Diego, California). Data are presented as mean ± SD, unless otherwise stated. Comparisons between two groups were performed using an unpaired two-tailed Student’s t test with Welch correction. Comparisons among multiple groups were analyzed by one-way repeated measures ANOVA followed by Tukey’s multiple-comparison test. Significant values are indicated by asterisks, where *p<0.05, **p<0, 01, ***p<0, 001, ****p<0, 0001.

## Results

### Intracellular Poly(I:C) induced inflammation and cell death in primary human macrophages

Human monocyte-derived macrophages (hMDMs) were exposed to the dsRNA mimetic Poly(I:C). Poly(I:C) is a synthetic analog of double stranded RNA (dsRNA) originally identified as a multistranded synthetic polynucleotide complex capable of inducing a robust interferon (IFN) response through its binding to pattern recognition receptors (PRRs) evolutionary conserved for natural dsRNA detection, including TLR3, MDA5, and RIG-I (14, 15, 40, 41). Poly(I:C) was added either in the culture medium or transfected using FuGENE, and inflammatory responses and cell death were evaluated. Transcription and release of IFN-β and TNF-α were measured after 6 h of Poly(I:C) stimulation as readout of IRF3- and NF-κB-driven responses, respectively. Transfected Poly(I:C) induced significant upregulation of IFN-β and TNF-α mRNA levels (**Figures 1A, B**) and increased cytokine release (**Figures 1C, D**). On the contrary, in the absence of transfection no IFN-β and TNF-α production was induced by Poly(I:C). To assess the contribution of transfected dsRNA to lytic cell death, membrane permeabilization and caspase-3 activation were monitored over time (**Figures 1E, F**). Membrane permeabilization and caspase-3 activation were observed starting at 7–8 h after stimulation. Consistently, LDH release was significantly increased at 18 h, but not at 6 h, in Poly(I:C)-transfected macrophages compared to controls (**Figure 1G**).

**Figure 1:**
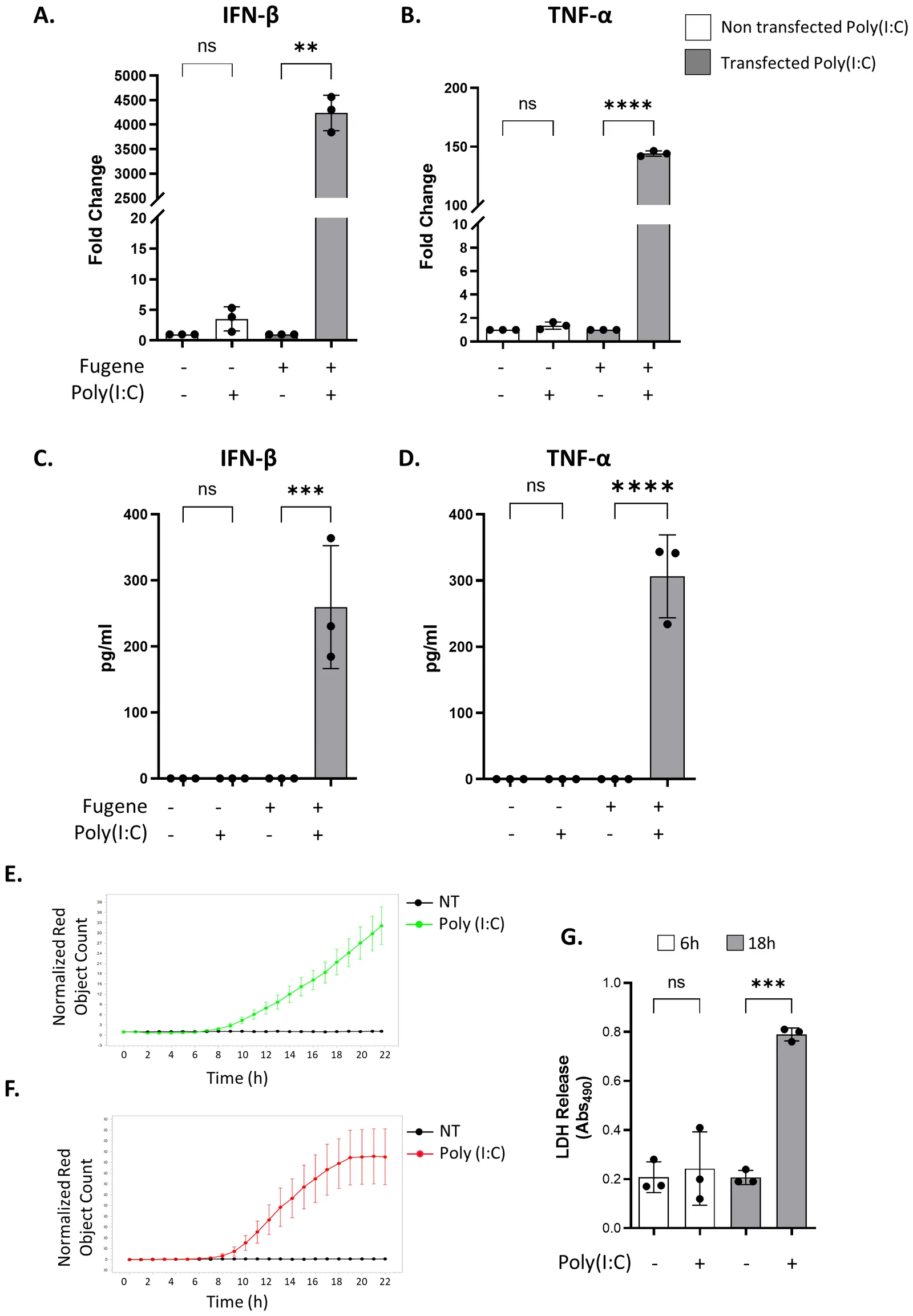
Intracellular Poly(I:C), activates inflammatory response and cell death. hMDMs were either stimulated with Poly(I:C) in the absence or presence of transfection reagent (10 μg/mL and 1 μg/mL, respectevely) using FuGENE^®^ as indicated. **(A, B)** After 6 h relative expression of IFN-β **(A)** and TNF-α **(B)** was assessed by RT-qPCR. Gene expression levels were normalized to the housekeeping gene GAPDH and expressed relative to untreated controls. **(C, D)** Release of IFN-β **(C)** and TNF-α **(D)** was quantified by ELISA. **(E, F)** Time-course analysis of plasma membrane permeabilization **(E)** and caspase-3/7 activation **(F)** in hMDMs transfected with Poly(I:C). Plasma membrane permeabilization was assessed by real-time uptake of SYTOX™ Green dye, while caspase-3/7 activation was monitored using the Incucyte^®^ Caspase-3/7 Dye for Apoptosis; both were quantified by live-cell imaging on the IncuCyte system. On the y-axis the object count (Green or Red) on mm2/phase area confluence normalized to T0 is reported. **(G)** LDH release measured in hMDMs after Poly(I:C) transfection at 6 h and 18 h. LDH activity was quantified by absorbance at 490 nm. Data are presented as mean ± SD (N = 3 independent donors). Statistical analysis was performed using one-way ANOVA. **P < 0.01, ***P<0.001, ****P<0.0001.

### Poly(I:C) induced caspase- and RIPK3-dependent lytic cell death

To explore the pathways leading to Poly(I:C)-induced cell death, we screened several inhibitors of apoptosis, necroptosis and pyroptosis alone or in combination (**Figure 2A**). Apoptotic and pyroptotic caspases were inhibited using the pan-caspase inhibitor Z-VAD-fmk (Z-VAD). Necroptosis was inhibited using the RIPK1 inhibitor Necrostatin-1 (Nec-1) or the RIPK3 inhibitor GSK872 (GSK). MCC950 was used to inhibit NLRP3-dependent pyroptosis. As reported in **Figure 2A**, Z-VAD and Nec-1 failed to inhibit cell death when added alone, but they reduced LDH release when used in combination. GSK alone reduced cell death, and the effect was not further modulated by the addition of Nec-1. Combined treatment with Z-VAD and GSK further reduced LDH release compared to the two treatments alone, and addition of Nec-1 had no effect. Cell death inhibition by Z-VAD/GSK combination was further confirmed by nuclei number count (**Supplementary Figure 3**). The NLRP3 inhibitor MCC950 failed to reduce LDH release, ruling out any involvement of NLRP3 in Poly(I:C)-induced cell death. This was consistent with the fact that Poly(I:C) did not induce significant upregulation of NLRP3 and Pro-IL-1β mRNA levels (**Supplementary Figure 4**). Overall, these data suggested that both apoptosis and RIPK3-dependent necroptosis contributed to Poly(I:C)-induced cell death.

**Figure 2:**
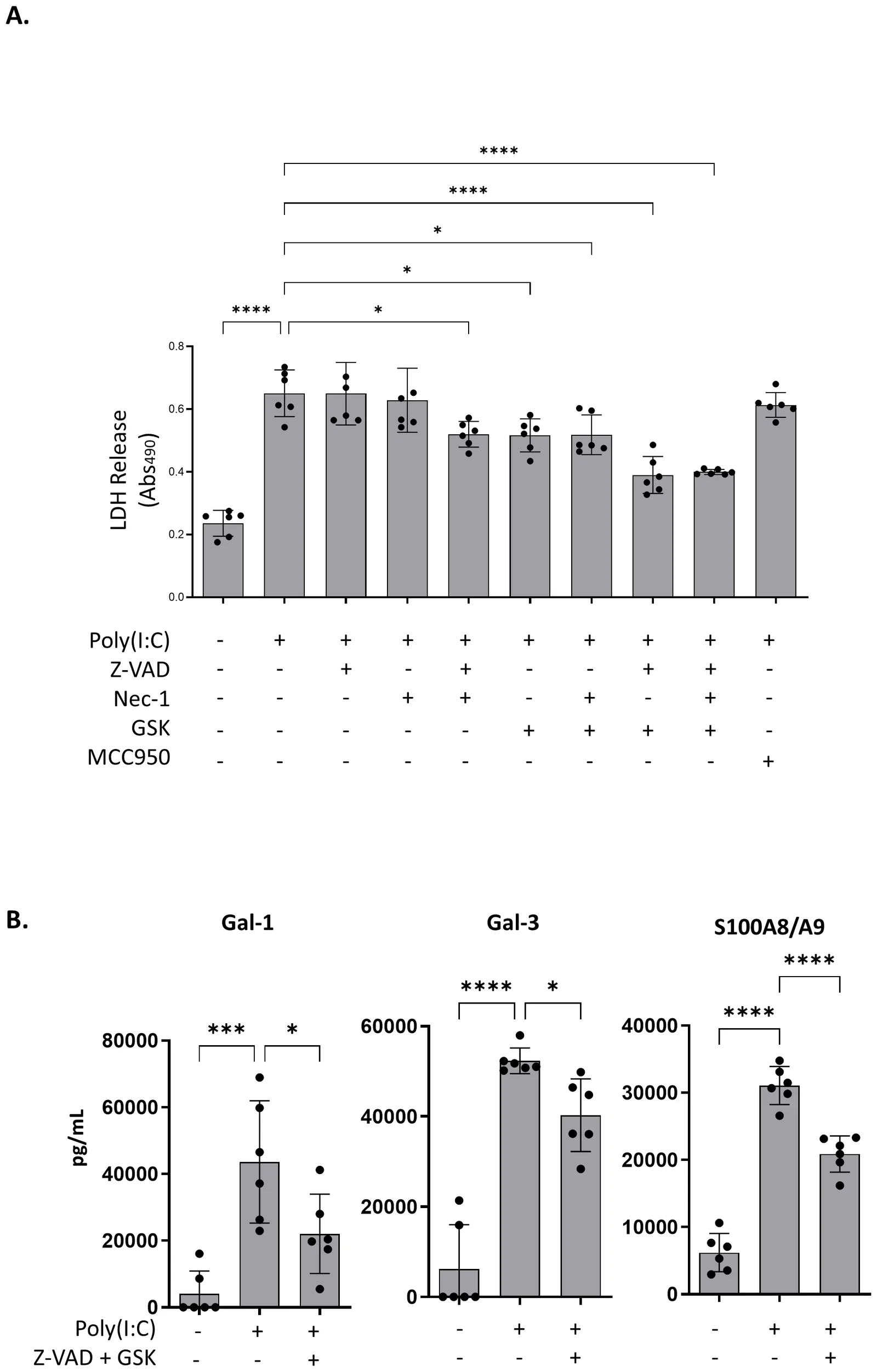
Inhibition of apoptotic and necroptotic pathways reduces Poly(I:C)-induced cell death and DAMPs release. hMDMs were pretreated for 1 h with 20 μM Z-VAD-FMK, 50 μM Necrostatin-1 (Nec-1), 10 μM GSK-872, or 1 μM MCC950, as indicated, followed by Poly(I:C) transfection (1 μg/mL) for 18 h. **(A)** LDH activity was measured in the supernatants. **(B)** Release of S100A8/A9, Galectin-3 (Gal-3) and Galectin-1 (Gal-1) was quantified in the supernatants by ELISA assay. Data are presented as mean ± SD (N = 3 independent donors), with technical replicates shown as individual data points. Statistical analysis was performed using one-way ANOVA. *P < 0.05, **P < 0.01, ***P<0.001, ****P<0.0001.

Lytic cell death is usually associated with DAMPs release (42). Among DAMPs, S100A8/A9 and Galectins (Gal) are emerging as key players in propagating inflammation during viral infections (43–45). Thus, the release of S100A8/A9, Gal-1 and Gal-3 was evaluated in hMDMs exposed to intracellular Poly(I:C), with or without cell death inhibitors GSK/Z-VAD (**Figure 2B**). We found that intracellular Poly(I:C) significantly induced the release of S100A8/A9, Gal-1 and Gal-3, and this effect was reverted by Z-VAD/GSK treatment.

### Intracellular Poly(I:C)-induced cell death exhibited apoptotic and necroptotic hallmarks

The impact of Z-VAD and GSK on cell death suggested that both apoptosis and necroptosis occurred in response to intracellular Poly(I:C) in hMDMs. To confirm these findings, key hallmarks of apoptosis and necroptosis were evaluated.

During apoptosis, nuclear chromatin becomes highly condensed, followed by DNA fragmentation and packaging into apoptotic bodies (28, 30). Additionally, mitochondrial membrane depolarization and loss of the electrochemical gradient occur (28, 29). We assessed nuclear DNA condensation and mitochondrial membrane potential (MMP) in hMDMs stimulated with intracellular Poly(I:C).

As shown in **Figure 3**, intracellular Poly(I:C) promoted nuclear condensation as indicated by a decrease of nuclear area and an increase of Hoechst fluorescence intensity (**Figures 3A–C**). Morphological alterations of nuclei were accompanied by loss of the MMP, as shown in **Figures 3D, E**. Activation of the apoptotic cascade in response to intracellular Poly(I:C) was confirmed by observing the cleavage of caspase-8 and -3 in response to Poly(I:C), which was completely blocked by Z-VAD (**Figures 4A, B**; **Supplementary Figure 5A**). In addition to TLR3/TRIF-mediated signaling (25, 27), dsRNA-induced cell death may involve additional pathways including RIPK3 (35). Aiming to assess RIPK3 contribution to caspase-8 activation in Poly(I:C)-treated hMDMs, the impact of selective inhibitor of RIPK3 (GSK) was evaluated and compared with the effects observed in the presence of a selective inhibitor of TRIF. We found that inhibition of both RIPK3 or TRIF reduced the activation of caspase-8/caspase-3 axis, and that combined TRIF and RIPK3 inhibition produced an additive suppressive effect, supporting cooperative pathway involvement (**Figures 4A, B**). We also observed RIPK3-dependent phosphorylation of MLKL in response to intracellular Poly(I:C), consistent with the activation of necroptotic cascade (**Figures 4C, D**).

**Figure 3:**
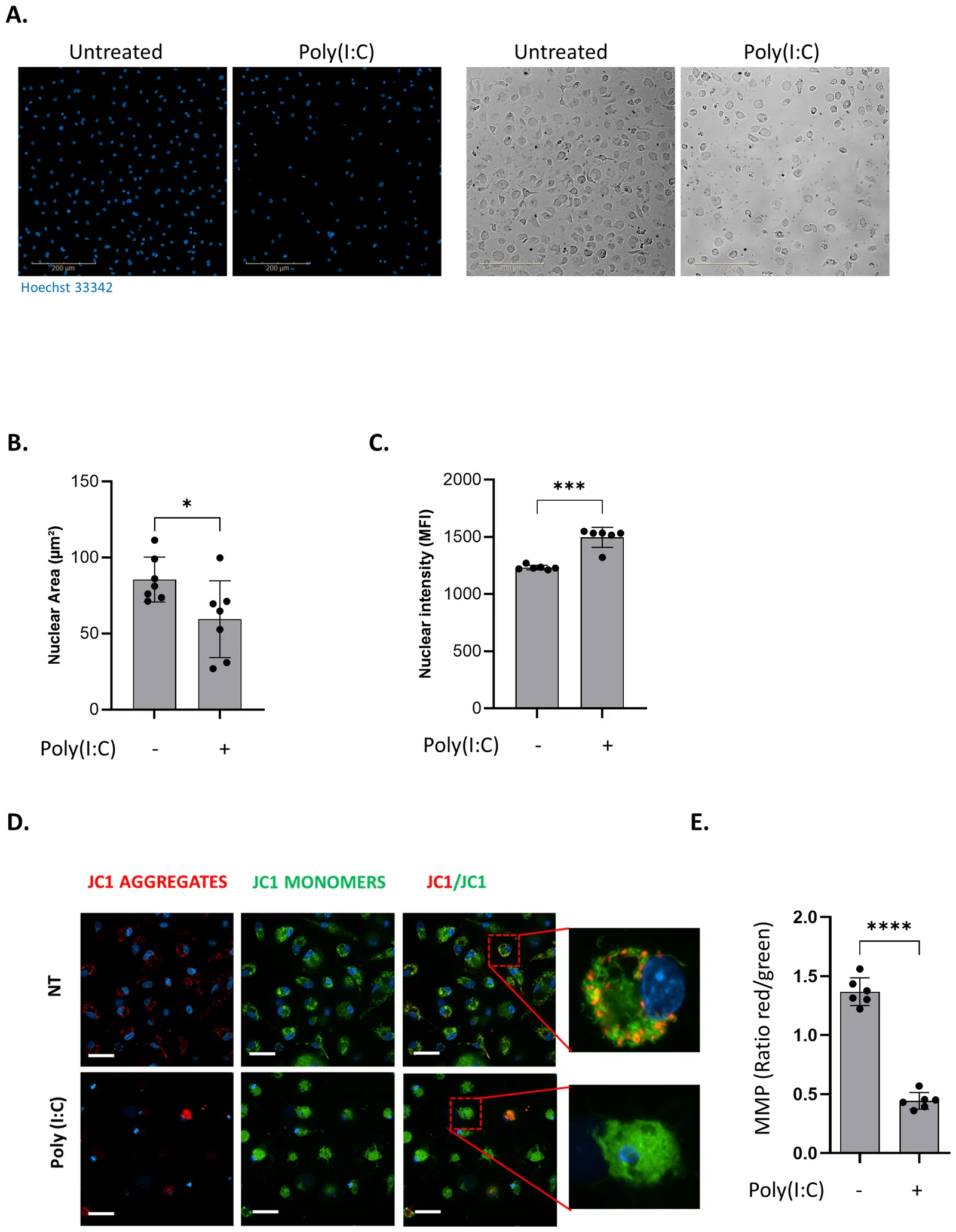
Poly(I:C)-transfected human macrophages display apoptotic hallmarks. hMDMs were transfected with Poly(I:C) (1 μg/mL) for 18 h. **(A)** Nuclei were counterstained with Hoechst 33342 and images were acquired using Operetta CLS (Revvity) with 40× objective. Corresponding brightfield images are reported on the right. Representative immunofluorescence images of one donor are reported. **(B)** Nuclear area and **(C)** nuclear fluorescence intensity were analyzed using the Harmony Software 4.9 (Revvity). **(D)** Mitochondrial membrane potential (MMP) was assessed using JC-1 dye. **(E)** Quantification of the red (485/590 nm) to green (485/529 nm) fluorescence intensity ratio measured by Spark Tecan plate reader. Images were acquired using a high-content imaging system (Operetta CLS, Revvity) using 40× - 63× objectives. Scale bar: 50 μm. Data in **(B, C, E)** are presented as mean ± SD (N = 3 independent donors), with technical replicates shown as individual data points. Statistical analysis was performed using an unpaired t-test. *P < 0.05, ***P<0.001, ****P<0.0001.

**Figure 4:**
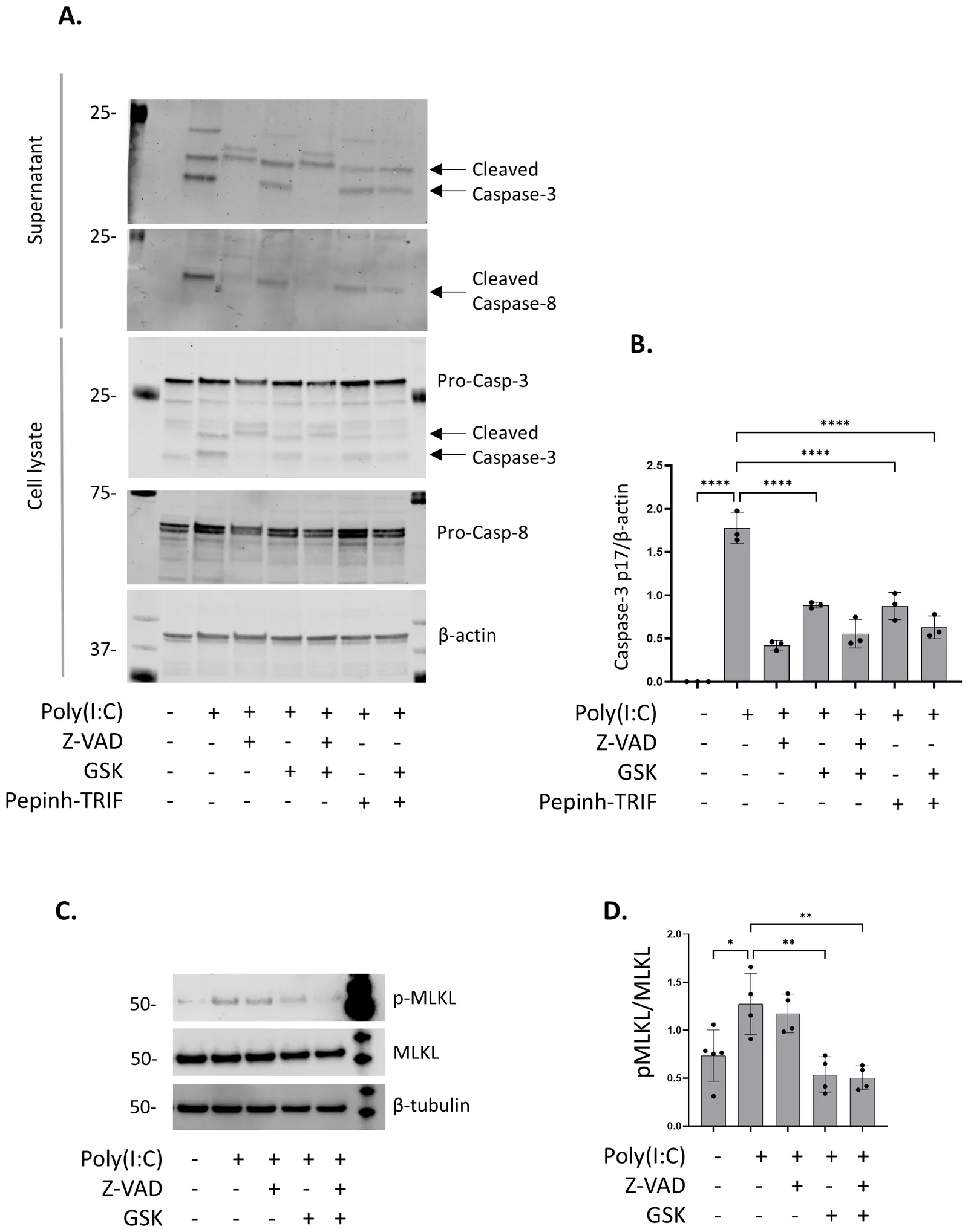
TRIF and RIPK3 control caspase activation and necroptotic signaling in Poly(I:C)-transfected macrophages. hMDMs were pretreated for 1 h with 20 μM Z-VAD-FMK, 10 μM GSK-872 and 25 μM Pepinh-TRIF, as indicated, followed by transfection with Poly(I:C) (1 μg/mL) for 18 h. **(A)** Representative western blot analysis of precipitated cell supernatants showing the cleavage of caspase-8 and caspase-3 in response to Poly(I:C) stimulation and its modulation by inhibition of TRIF and RIPK3. Corresponding cell lysates were immunoblotted for total caspase-8, total and cleaved caspase-3. β-actin was used as a loading control. **(B)** Densitometric analysis of cleaved caspase-3 (p17 band) in cell lysates, normalized to β-actin. **(C)** Western blot analysis of cell lysates showing phosphorylation of MLKL. **(D)** Densitometric analysis of p-MLKL levels in cell lysates, normalized to MLKL. One representative western blot image from N = 3 (N = 4 for pMLKL/MLKL) independent donors is reported. Data are reported as mean ± SD. *P < 0.05, **P<0.01, ****P<0.0001.

### Intracellular Poly(I:C) induced GSDME activation, while leading to inactivation of GSDMD

Since intracellular Poly(I:C) activated the caspase-8/caspase-3 axis, and it is known that caspase-3 cleaves gasdermin E (GSDME) at Asp270 to generate the active N-terminal fragment (31, 32, 34), we examined GSDME processing. Intracellular Poly(I:C) induced cleavage of GSDME (GSDME-NT), and this effect was reverted by treatment with Z-VAD/GSK (**Figures 5A, B**; **Supplementary Figure 5B**). Confocal imaging further confirmed the appearance of the N-terminal fragment of GSDME-NT in response to intracellular Poly(I:C), which was reduced by Z-VAD/GSK treatment. GSDME-NT appeared to be localized in the intracellular compartment and not in the plasma membrane (**Figures 5C, D**). Recent reports have shown that GSDME can localize in mitochondria enhancing caspase-3 activation during apoptosis (46). Therefore, we investigated by confocal immunofluorescence analysis whether GSDME-NT co-localized with the mitochondrial protein TOM20 in Poly(I:C)-stimulated hMDMs. Data reported in **Figure 5E** confirmed co-localization of GSDME-NT with TOM20, indicating mitochondrial localization. We next investigated the functional consequences of mitochondrial GSDME localization by looking at caspase-3 activation. To this end, we generated GSDME knockdown macrophages. As shown in **Figure 5F**, Poly(I:C)-induced caspase-3 activation was significantly reduced in GSDME-knockdown THP-1 macrophages compared with scramble control cells, supporting a role for GSDME in amplifying caspase-3 activation (**Figures 5F, G**).

**Figure 5:**
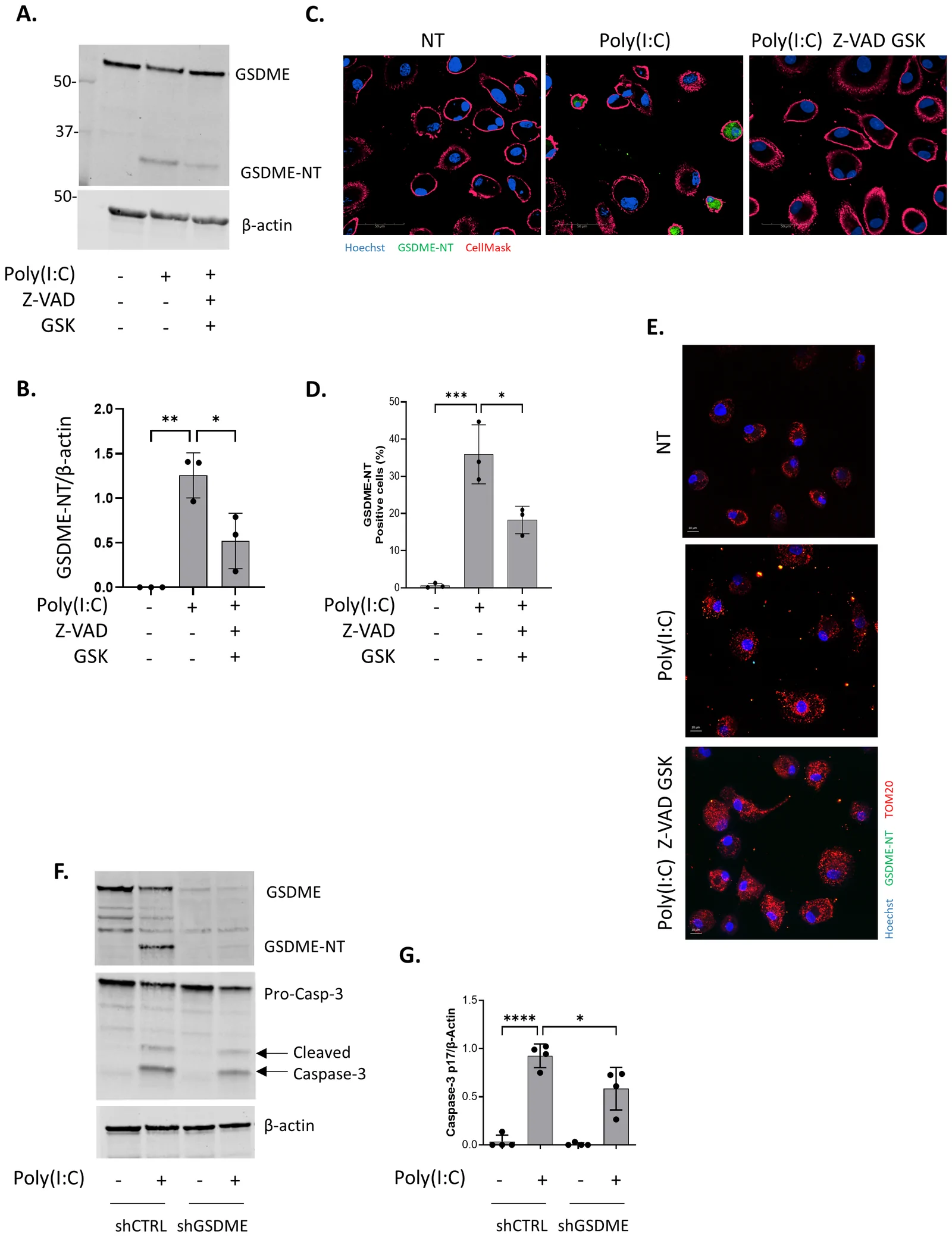
Intracellular Poly(I:C) induced cleavedGSDME localized in mitochondria and enhanced caspase-3 activation. hMDMs were pretreated with 20 μM Z-VAD-FMK and 10 μM GSK-872 for 1 h, followed by transfection with Poly(I:C) (1 μg/mL) for 18 h. **(A)** Representative western blot analysis of cell lysates showing full-length GSDME and the cleaved N-terminal fragment (GSDME-NT). One representative western blot image from N = 3 independent donors is reported. **(B)** Densitometric analysis of cleaved GSDME (GSDME-NT) in cell lysates, normalized to β-actin. **(C)** Immunofluorescence analysis of hMDMs stained with Hoechst 33342 (blue), CellMask™ plasma membrane marker (red), and an antibody specifically recognizing GSDME-NT (green). Images were acquired using a high-content imaging system (Operetta CLS, Revvity) with a 63× objective. Scale bar: 50 μm. **(D)** Quantification of the percentage of GSDME-NT positive cells. Analysis was performed using the Harmony Software 4.9 (Revvity). Data are presented as mean ± SD (N = 3 independent donors). Statistical analysis was performed using one-way ANOVA. ****P<0.0001. **(E)** Immunofluorescence analysis of hMDMs stained with Hoechst 33342 (blue) and antibodies specifically recognizing GSDME-NT (green) e TOM20 (red). Images were acquired using Nikon Crest X-light V3 spinning disk confocal microscope with 100x oil objective. Scale bar: 10 μm. One image representative of N = 3 independent donors is reported. **(F)** THP1 macrophages knocked down for GSDME and scramble controls were transfected with Poly(I:C) (1µg/ml, 18 h) and caspase-3 cleavage was evaluated by western blot. One representative western blot image from N = 4 independent experiments is reported. **(G)** Densitometric analysis of cleaved caspase-3 (p-17) in cell lysates, normalized to β-actin. Data are expressed as mean ± SD.

Caspase-3 also cleaves GSDMD at D87 in the N-terminal domain, generating an inactive fragment of 43 kDa (47). Consistently, we found that intracellular Poly(I:C) induced the formation of GSDMD inactive form which disappeared following Z-VAD treatment (**Supplementary Figure 6**).

### RIPK3 acted as key mediator of inflammation in response to intracellular Poly(I:C) in hMDMs

Data so far reported indicated that RIPK3 and TRIF jointly contributed to the activation of the caspase-8/caspase-3 axis and that RIPK3 was a regulator of both the apoptotic and necroptotic cascade. We next explored the involvement of these two signalling axes on the activation of the proinflammatory response. To this purpose, the release of IFN-β and TNF-α was measured at 6 h and 18 h after intracellular Poly(I:C) stimulation in the presence of the inhibitor of RIPK3 (GSK) or TRIF (Pepinh-TRIF), alone or in combination. As shown in **Figure 6**, release of IFN-β and TNF-α was significantly enhanced after 6h from Poly(I:C) stimulation and further increased at 18h. Early cytokine induction was effectively suppressed by RIPK3 inhibition, whereas TRIF inhibition showed no significant effect at 6 h (**Figure 6A**). TRIF-dependent contribution became apparent only at later time points, as partial inhibition of IFN-β and TNF-α responses emerged at 18 h. When GSK and Pepinh-TRIF were added in combination, no additional inhibition was observed compared to GSK alone (**Figure 6B**). It has been reported that the expression of the dsRNA receptors TLR3 and MDA5 can be further increased in response to IFN signaling following Poly(I:C) stimulation (48–52). Consistent with this, we observed a marked upregulation of both TLR3 and MDA5 protein levels in response to Poly(I:C). This effect was reverted by RIPK3 inhibition. On the contrary, TRIF inhibition did not display any effect on Poly(I:C)-induced upregulation of MDA5 and TLR3 (**Figures 6C, D**).

**Figure 6:**
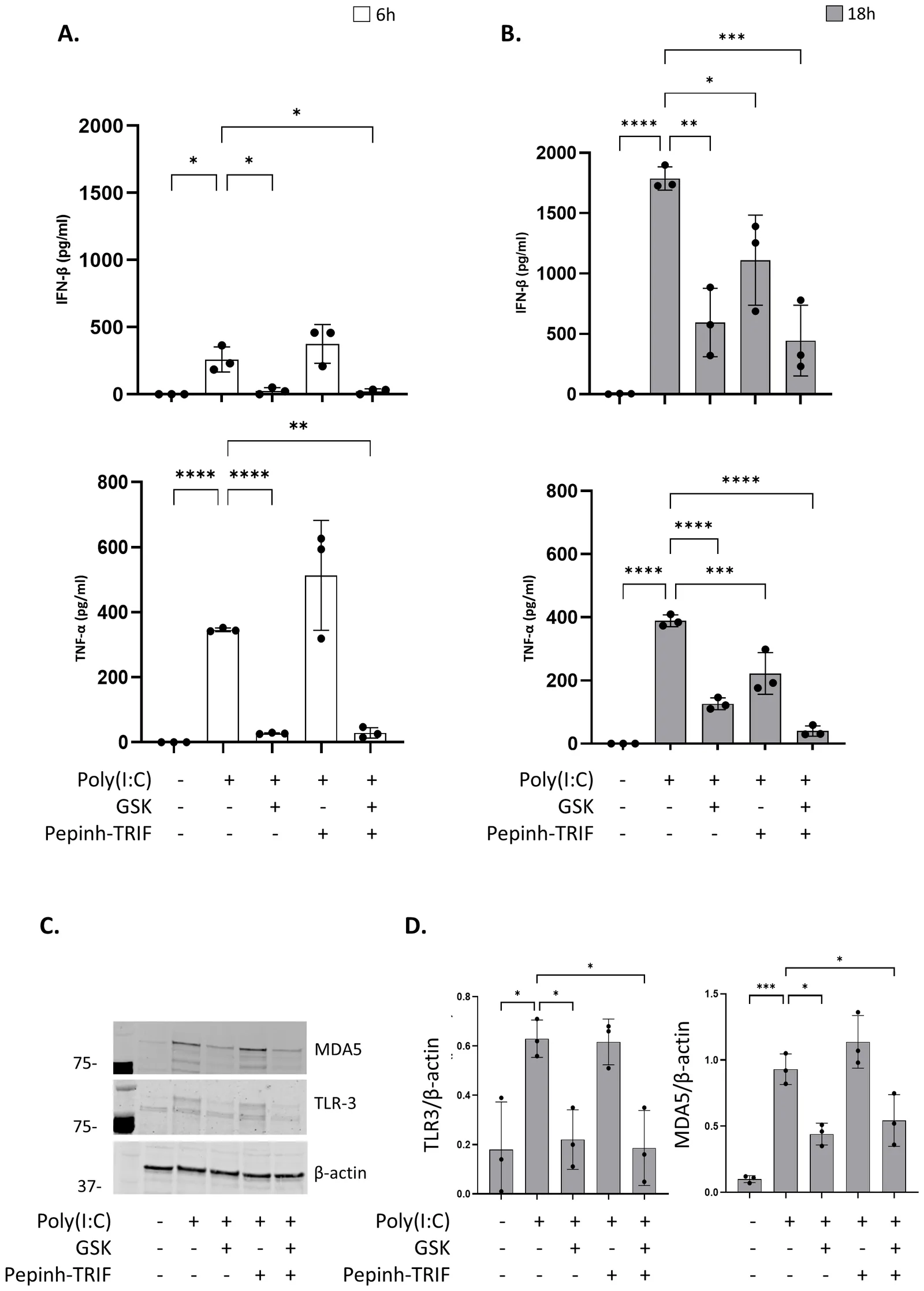
RIPK3 controls the early inflammatory response after intracellular Poly(I:C) stimulation and regulates TLR3 and MDA5 induction. hMDMs were pretreated with 10 μM GSK-872 and 25 μM Pepinh-TRIF, alone or in combination for 1 h, followed by transfection with Poly(I:C) (1 μg/mL) **(A, B)** Release of TNF-α and IFN-β was quantified by ELISA assay at 6 h **(A)** and 18 h **(B)**. Data are presented as mean ± SD from N = 3 independent donors. **(C)** Expression of TLR3 and MDA5 was measured after 18 h. β-actin was used as a loading control. One representative western blot image from N = 3 independent donors is reported. **(D)** Densitometric analysis of TLR3 and MDA5 levels in cell lysates, normalized to β-actin. Data are presented as mean ± SD (N = 3 independent donors). Statistical analysis was performed using one-way ANOVA. *P < 0.05, **P < 0.01, ***P<0.001, ****P<0.0001.

## Discussion

Recognition of dsRNA by MDA5 and TLR3 activates MAVS- and TRIF-dependent signaling cascades, respectively, leading to inflammatory cytokine release and caspase-8-dependent apoptosis (14, 22, 25, 53). It has been reported that the kinase RIPK3 participates to both the inflammatory response and cell death regulated programs during viral infections (23, 35, 54, 55). However, what is the role of RIPK3 in dsRNA-induced cell death and inflammatory response and what is the connection between RIPK3- and TLR3/TRIF-dependent signaling axes remain to be elucidated. Our findings show that Poly(I:C)-induced inflammatory response occurs early in time and is regulated by RIPK3, which appears to be dominant over the TLR3/TRIF axis, which becomes activated at later timepoints. We also show that in response to intracellular Poly(I:C) macrophages undergo a mixed form of regulated cell death where both apoptosis and necroptosis occur, with RIPK3- and TRIF-mediated axes jointly contributing to caspase-8 activation and with RIPK3 being the regulator of the necroptotic cascade.

Our data show that Poly(I:C) internalization is required to trigger both the inflammatory response and cell death, with the latter occurring at later timepoint. These findings are consistent with the notion that intracellular dsRNA sensors, such as TLR3 and MDA-5, are critical for initiating antiviral responses in human macrophages (12, 14, 20). Our data reveal that intracellular Poly(I:C) induces a mixed form of programmed cell death characterized by the simultaneous engagement of apoptotic and necroptotic pathways. Consistently, only the combined inhibition of caspases and RIPK3 provided significant protection from cell death. Additionally, we show that intracellular Poly(I:C)-induced cell death does not depend by NLRP3 activation as dsRNA did not act as priming signal. The functional relevance of lytic cell death program was further supported by the release of DAMPs, including S100A8/A9 and galectins, which are known amplifiers of inflammatory responses (43–45).

It is well established that intracellular dsRNA sensing by TLR3 leads to caspase-8/caspase-3 axis activation through recruitment of the adaptor protein TRIF (25–27) and our data are consistent with this model. In addition, our findings indicate that RIPK3 also contributes to dsRNA-induced activation of caspase-8/caspase-3 axis activation through a pathway that operates in parallel to TRIF-mediated signaling. Since it has been reported that RIPK3 may interact with MAVS complex (23), assembled following the sensing of Poly(I:C) by MDA5 (13, 14), RIPK3-dependent caspase-8/caspase-3 axis activation may be initiated downstream of MDA5. Alternatively, activation of RIPK3 could occur downstream of other canonical upstream regulators such as RIPK1 or ZBP1 (56, 57), known to engage RIPK3 in response to nucleic acid sensing. Consistent with the activation of necroptosis, we also observed RIPK3-dependent phosphorylation of MLKL, confirming that intracellular Poly(I:C) engages canonical necroptotic machinery in human macrophages. Together, these data position RIPK3 as a central signaling hub that integrates apoptotic and necroptotic cues in response to intracellular dsRNA. Future studies using RIPK3 genetic loss-of-function macrophages will be instrumental in further dissecting the molecular mechanisms by which RIPK3 regulates cell death and inflammatory signaling in response to intracellular dsRNA.

Additionally, we found that GSDME was cleaved to its N-terminal fragment in response to intracellular Poly(I:C). However, despite it has been reported that GSDME-NT may localize to plasma membrane in neutrophils or tumor cells (58, 59), what is the role of cleaved GSDME in macrophages is still unclear. We show that GSDME-NT remained within the intracellular compartment and localized in the mitochondria contributing to caspase-3 activation, consistent with previous reports (46).

In addition to cell death, the inflammatory process is another major response induced by intracellular dsRNA receptors, which lead to the expression and release of type I IFNs and NF-kB –regulated cytokines (10–15, 18, 20, 23). However, how inflammation interfaces with the execution of cell death and the temporal dynamics of the two events remain unclear. Here, we show that the inflammatory response precedes cell death. Our data also suggest that RIPK3 regulates the early inflammatory response, and precedes the subsequent involvement of the TLR3/TRIF axis. This may be consistent with RIPK3 acting downstream of MDA5/MAVS (23), whose signaling has been reported to be dominant over TLR3 in the control of type I IFNs production (14, 60). Our data indicate that TLR3 becomes involved at later time points. This may be due to the different subcellular localization of the sensors, as TLR3 requires the internalization of dsRNA into endosomal compartments (61–63), Of note, RIPK3 appears to be a key regulator for both IFN-β and TNF-α, suggesting a role in controlling both IRF3- and NF-kB-dependent inflammatory responses. It has been reported that type I IFNs induce upregulation of MDA5 and TLR3 (48–52). This is consistent with the upregulation of the two receptors that we observed after intracellular Poly(I:C) treatment and that may further enhance the inflammatory response and sustain the activation of cell death. Collectively, our findings identify RIPK3 as a key integrator of inflammation and cell death in human macrophages responding to intracellular dsRNA. By coordinating cytokine production, apoptotic and necroptotic signaling, RIPK3 emerges as a central node in antiviral macrophage responses, with potential implications for the pathogenesis and therapeutic targeting of viral-induced inflammatory diseases.

## Data Availability

The original contributions presented in the study are included in the article/**Supplementary Material**. Further inquiries can be directed to the corresponding author.
