# Supplementary material for: Human macrophages activate a coordinated program linking inflammation and cell death in response to intracellular Poly(I:C)
Source: Front Immunol. 2026 Oct 2;17:1827486. doi: 10.3389/fimmu.2026.1827486 (PMC13672200; doi:10.3389/fimmu.2026.1827486)
Supplement: Supplementary file 1 [file DataSheet1.pdf]

Supplementary Information

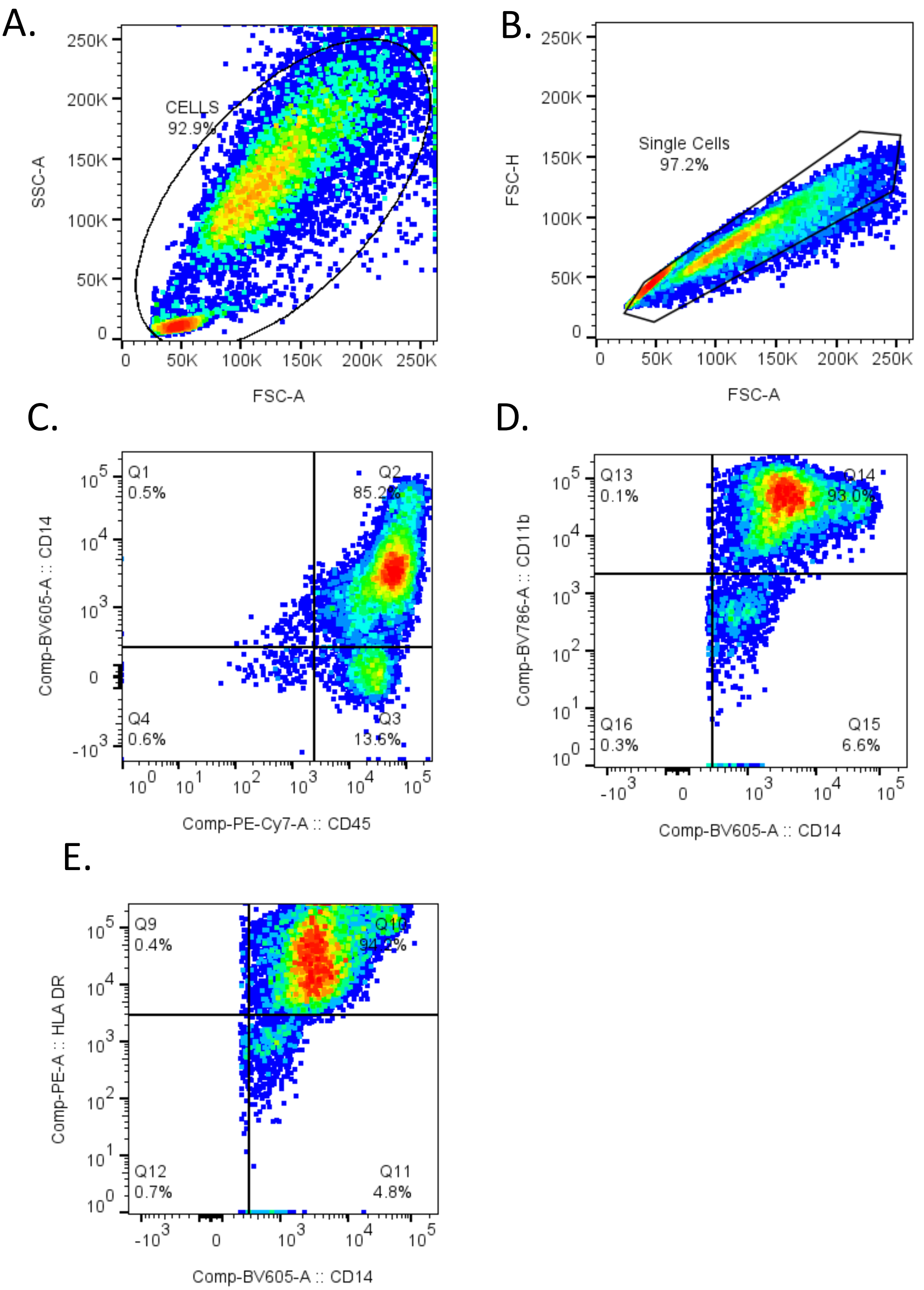

Figure S1. Flow cytometric characterization of human monocyte-derived macrophages. Human monocyte-derived macrophages (hMDM) were generated from peripheral blood mononuclear cells cultured complete RPMI1640 medium supplemented with 10% FBS and 50 ng/ml of human M-CSF. After 7 days, cells were harvested and characterized by flow cytometry. Macrophages were stained with anti-CD14 (BD Biosciences, cat. 564054), anti-CD45 (BD Biosciences, cat. 557748), anti-CD11b (BD Biosciences, cat. 742642) and anti-HLA-DR (Miltenyi Biotec, cat. 130-111-789) according to the manufacturer's instructions. Representative gating strategy is shown in density plots. (A, B) Cells were first gated on singlets (FSC-H vs FSC-A) (C). Leukocytes were identified as $CD45^+$ events and Macrophages were subsequently defined based on CD14 expression. All downstream analyses were performed within the $CD14^+$ population. (D) Representative density plots showing co-expression of CD11b within the $CD14^+$ gate, confirming myeloid identity and successful differentiation into macrophages. (E) Representative density plots of HLA-DR expression within $CD14^+$ cells, indicating antigen-presenting capacity of differentiated macrophages. Data were acquired using a BD FACSCelesta™ flow cytometer (BD Biosciences) and analyzed with BD FACSDiva™ software. One representative plot of three independent donors is reported.

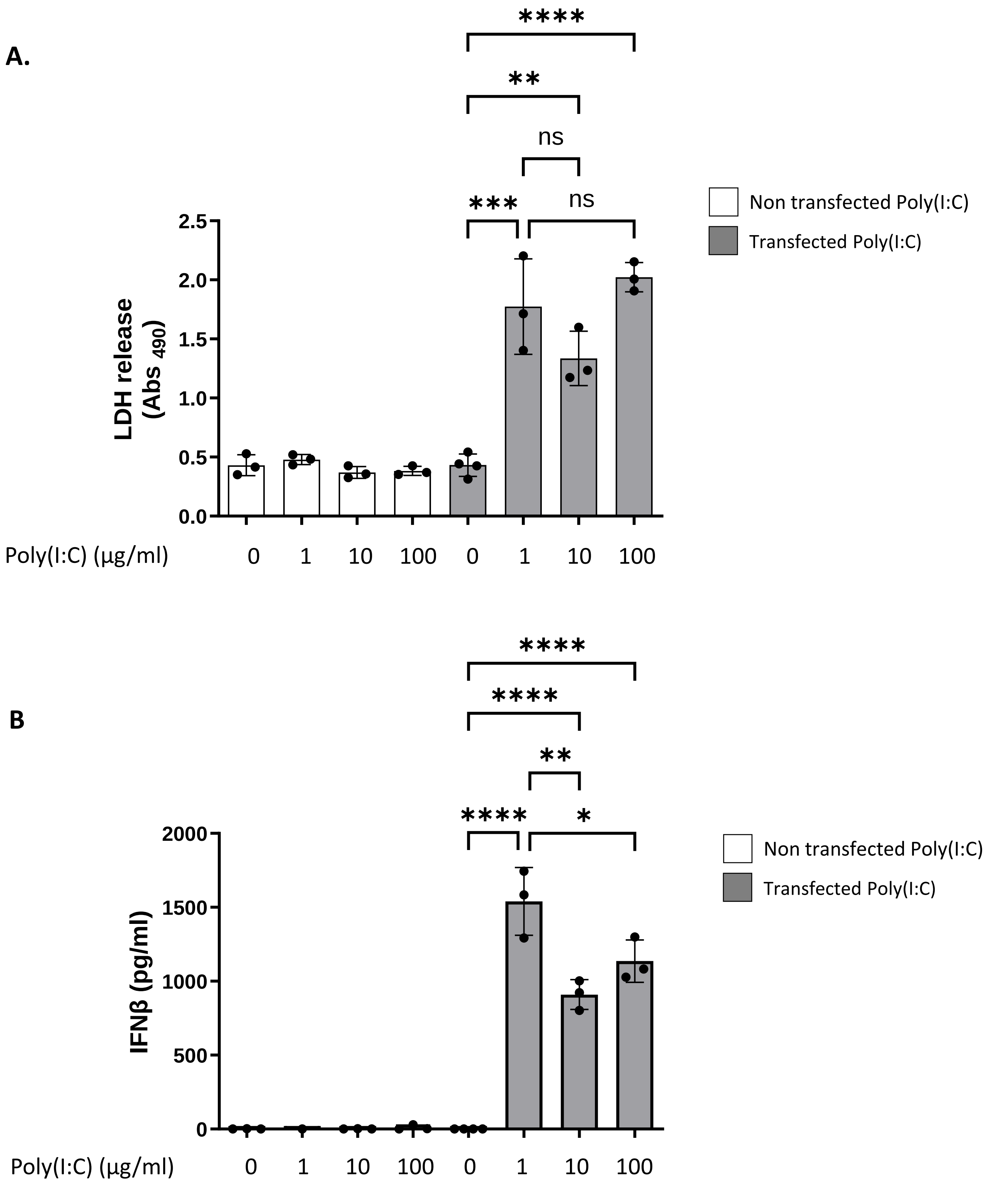

Figure S2. Dose-response stimulation of hMDMs with non-transfected or transfected Poly(I:C). hMDMs were stimulated with the indicated concentration of Poly(I:C) in the absence or presence of FuGENE® transfection reagent. Supernatants were collected after 18h. (A) LDH release was measured in the supernatants (B) Release of IFN-β was quantified by ELISA. Data are presented as mean ± SD (N=3 independent donors). Statistical analysis was performed using one-way ANOVA. *P < 0.05, **P < 0.01, ***P<0.001, ****P<0.0001.

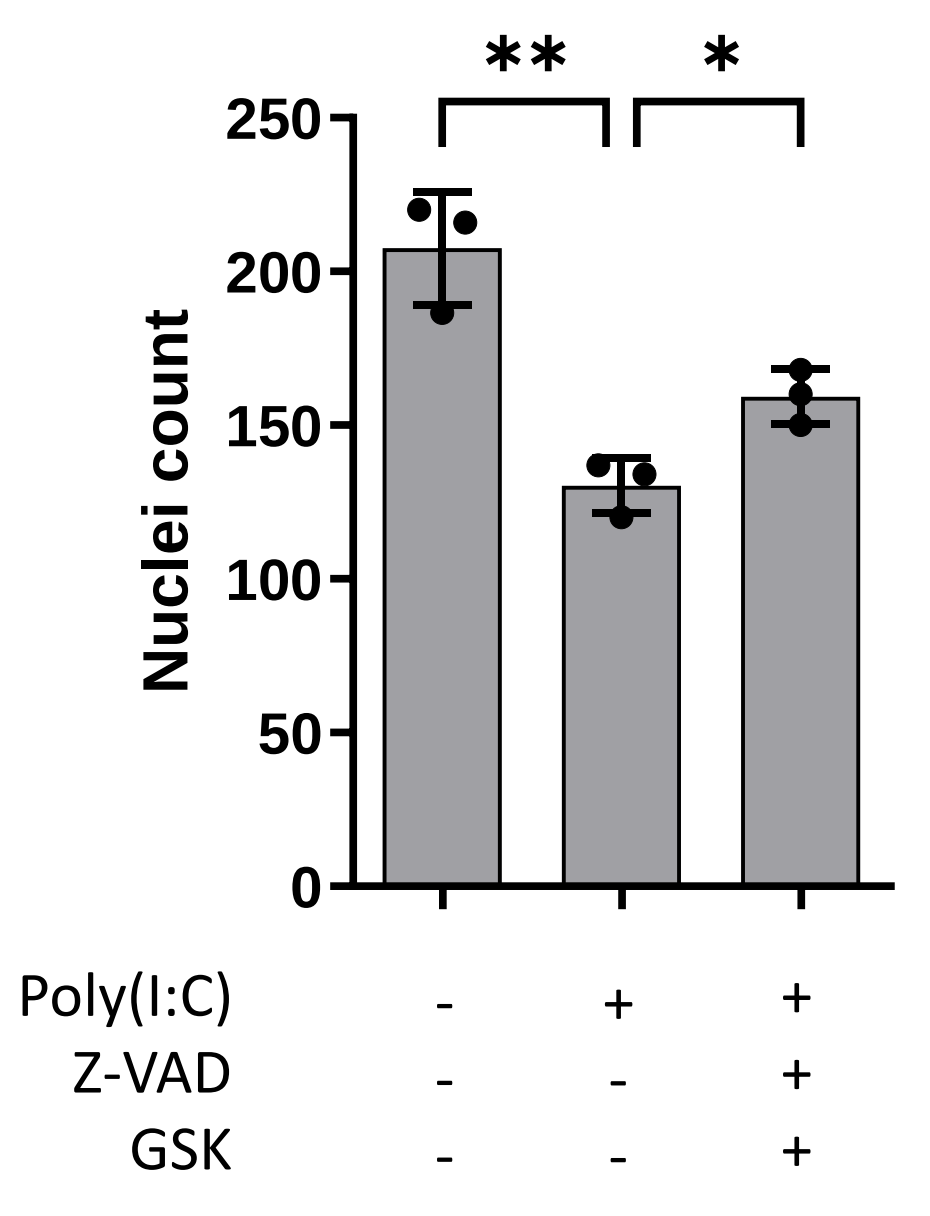

Figure S3. Z-VAD+GSK treatment reduces Poly(I:C)-induced cell death. hMDMs were pretreated for 1 h with 20 µM Z-VAD-FMK (Z-VAD) and 10 µM GSK-872 (GSK) followed by Poly(I:C) transfection (1 µg/mL) for 18 h. Nuclei were counterstained with Hoechst 33342 and images were acquired using Operetta CLS (Revvity) with 40× objective. Nuclei were counted using the Harmony Software 4.9 (Revvity). Statistical analysis was performed using one-way ANOVA. *P < 0.05, **P < 0.01.

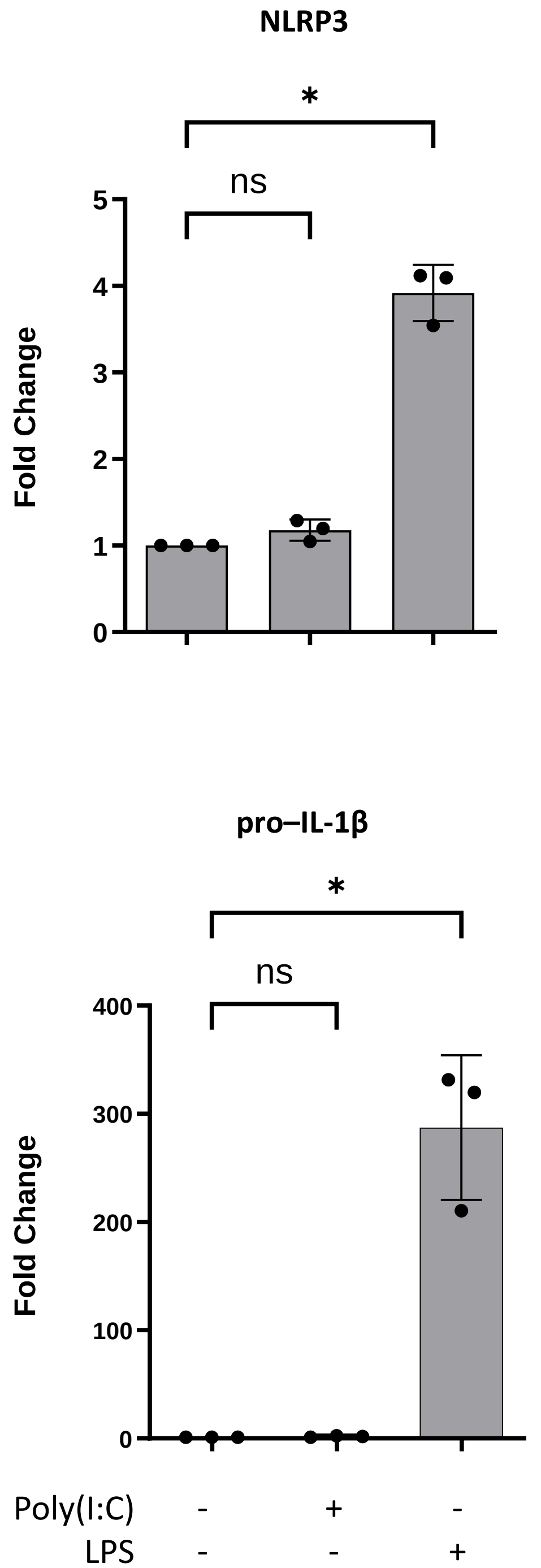

Figure S4. Poly(I:C) does not induce priming of NLRP3 and pro–IL-1β in human macrophages.
hMDMs were transfected with Poly(I:C) (1 µg/mL) using FuGENE® transfection reagent and incubated for 18 h, or stimulated with LPS (1 µg/mL - L3012 Sigma) for 3 h, as indicated. Relative expression of NLRP3 and pro–IL-1β was assessed by RT–PCR using specific FAM-labeled probe and primers (prevalidated TaqMan Gene expression assay for NLRP3 (Hs00918082-m1) and pro-IL-1β (Hs01555410-m1), Applied Biosystems). Gene expression levels were normalized to the housekeeping gene GAPDH and expressed as fold change relative to untreated controls. Data are presented as mean ± SD (N = 3 independent donors). Statistical analysis was performed using one-way ANOVA followed by Tukey's multiple-comparison test. *P < 0.05

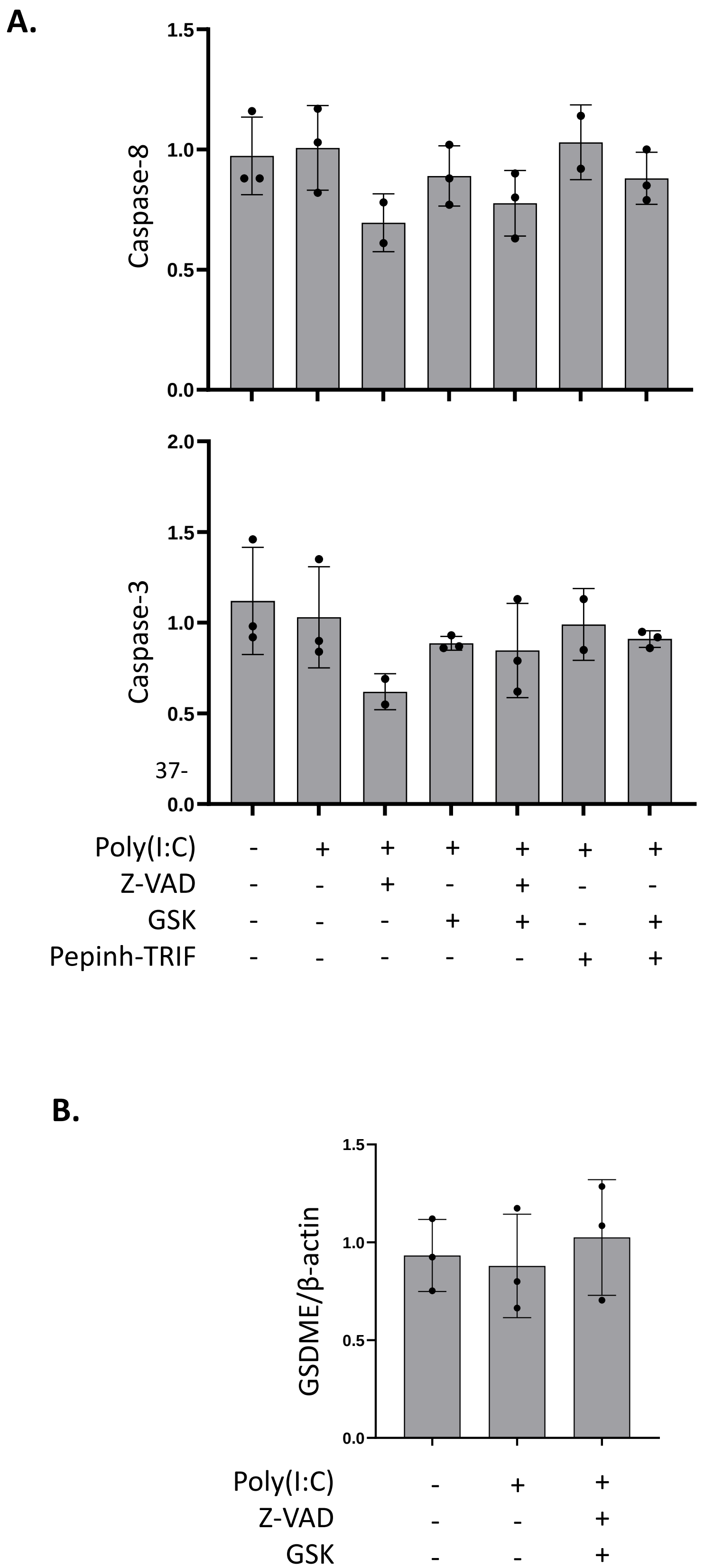

Figure S5. Densitometric analysis of full length caspase-8, caspase-3 from Figure 4A **(A)** and GSDME full length from figure 5A **(B)**, normalized to β-actin. Data are presented as mean ± SD (N = 3 independent donors).

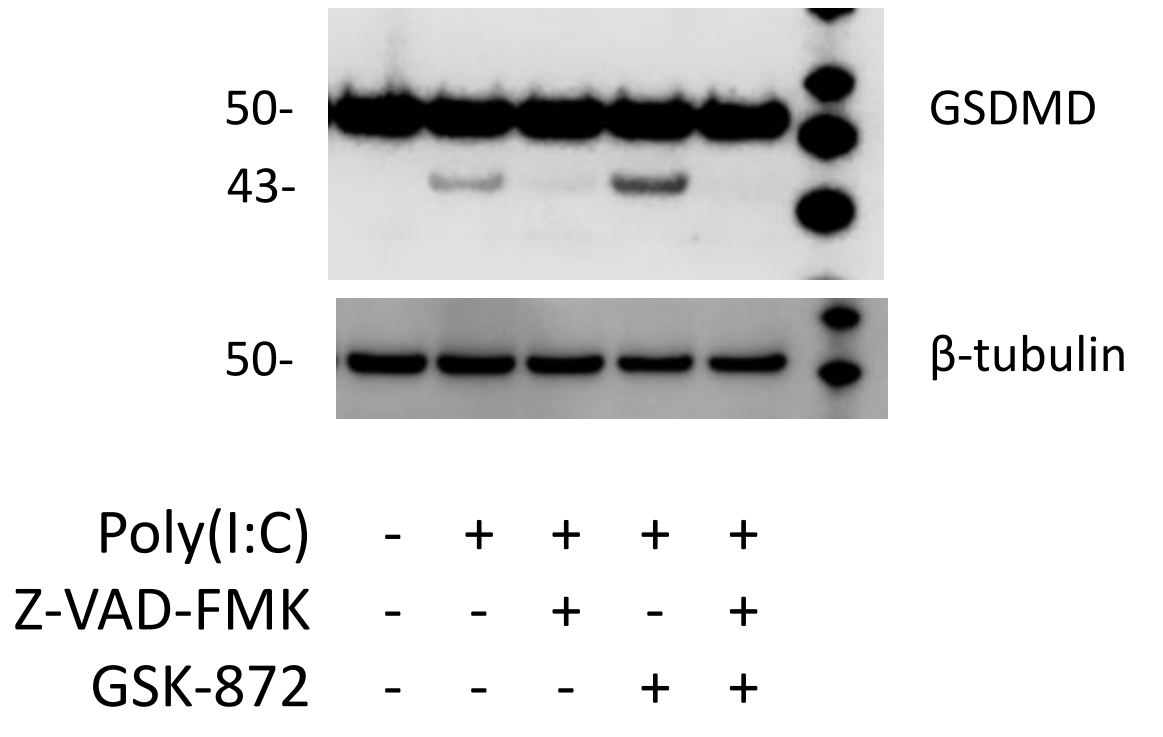

Figure S6. The inactive p43 fragment of gasdermin D is generated in human macrophages transfected with Poly(I:C).

hMDMs were transfected with Poly(I:C) (1 µg/mL) using FuGENE® transfection reagent and incubated for 18 h. Representative Western blot analysis of cell lysates showing full-length gasdermin D (GSDMD) and the p43 cleaved fragment. β-tubulin was used as a loading control. One representative western blot image from N=3 independent donors is reported.
